# (*E*)-*N*′-(4-Chloro­benzyl­idene)-3,4,5-trimethoxy­benzohydrazide

**DOI:** 10.1107/S1600536808039044

**Published:** 2008-11-26

**Authors:** Yu-Min Wang, Zhen-Dong Zhao, Yu-Xiang Chen, Liang-Wu Bi

**Affiliations:** a Institute of Chemical Industry of Forest Products, Chinese Academy of Forestry, Nanjing 210042, People’s Republic of China

## Abstract

The title compound, C_17_H_17_ClN_2_O_4_, was synthesized from 3,4,5-trimethoxy­benzohydrazide and 4-chloro­benzaldehyde. In the crystal structure, packing is stabilized by intramolecular C—H⋯O and inter­molecular N—H⋯O and C—H⋯O hydrogen-bonding inter­actions.

## Related literature

For related literature, see: Yang *et al.* (1996 ▶); Nawar *et al.* (2000 ▶); Gardner *et al.* (1991 ▶); Labouta *et al.* (1989 ▶); Wang *et al.* (2008 ▶); Allen *et al.* (1987 ▶).
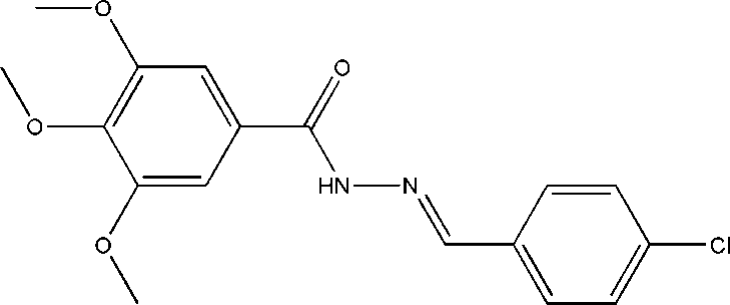


## Experimental

### 

#### Crystal data

C_17_H_17_ClN_2_O_4_
*M* *_r_* = 348.78
Triclinic, 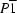

*a* = 5.119 (2) Å
*b* = 8.210 (4) Å
*c* = 20.276 (9) Å
α = 101.055 (7)°
β = 92.362 (7)°
γ = 101.459 (7)°
*V* = 816.9 (7) Å^3^
*Z* = 2
Mo *K*α radiation
μ = 0.26 mm^−1^
*T* = 273 (2) K
0.12 × 0.10 × 0.06 mm

#### Data collection

Bruker APEX CCD area-detector diffractometer
Absorption correction: multi-scan (*SADABS*; Sheldrick, 1996 ▶) *T*_min_ = 0.970, *T*_max_ = 0.985
4307 measured reflections
2860 independent reflections
2497 reflections with *I* > 2σ(*I*)
*R*_int_ = 0.017

#### Refinement

*R*[*F* ^2^ > 2σ(*F* ^2^)] = 0.046
*wR*(*F* ^2^) = 0.128
*S* = 1.03
2860 reflections
218 parameters
H-atom parameters constrained
Δρ_max_ = 0.59 e Å^−3^
Δρ_min_ = −0.39 e Å^−3^

### 

Data collection: *SMART* (Bruker, 1997 ▶); cell refinement: *SAINT* (Bruker, 1997 ▶); data reduction: *SAINT*; program(s) used to solve structure: *SHELXS97* (Sheldrick, 2008 ▶); program(s) used to refine structure: *SHELXL97* (Sheldrick, 2008 ▶); molecular graphics: *SHELXTL* (Sheldrick, 2008 ▶); software used to prepare material for publication: *SHELXTL*.

## Supplementary Material

Crystal structure: contains datablocks I, global. DOI: 10.1107/S1600536808039044/at2683sup1.cif

Structure factors: contains datablocks I. DOI: 10.1107/S1600536808039044/at2683Isup2.hkl

Additional supplementary materials:  crystallographic information; 3D view; checkCIF report

## Figures and Tables

**Table 1:** Hydrogen-bond geometry (Å, °)

*D*—H⋯*A*	*D*—H	H⋯*A*	*D*⋯*A*	*D*—H⋯*A*
N2—H2⋯O1^i^	0.86	2.18	2.943 (3)	147
C8—H8*C*⋯O4	0.96	2.26	2.896 (4)	123
C11—H11⋯O1^i^	0.93	2.43	3.145 (3)	134
C16—H16⋯O1^ii^	0.93	2.57	3.368 (3)	144

Symmetry codes: (i) 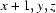; (ii) 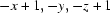.

**Fig. 1.**
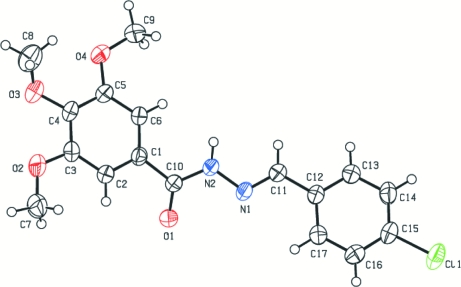
A view of the molecular structure of (I), showing displacement ellipsoids at the 50% probability level.

**Fig. 2.**
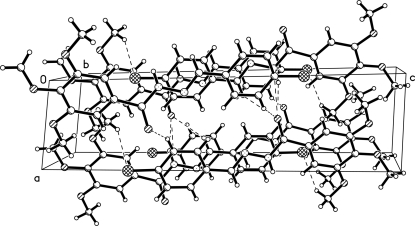
Packing diagram of the title structure.

## supplementary crystallographic information

### Comment

3,4,5-Trimethoxybenzohydrazide and their deviatives show moderate fungicidal and anti-bacterial activities (Gardner *et al.*, 1991). The antibacterial activity of formylhydrazines and formylhydrazones has been reported by Labouta *et al.* (1989). Many derivatives of formylhydrazines have interesting biological properties. So we synthesized several derivatives of 3,4,5-trimethoxybenzohydrazide. In our previous paper we have reported the crystal structure of (*E*)—*N*'-(2-hydroxybenzylidene)-3,4,5-trimethoxybenzohydrazide (Wang *et al.*, 2008). Now we synthesized the title compound (I) and report here its crystal structure.

The molecular structure of (I) is shown in Fig. 1. All bond lengths and angles in (I) are normal (Allen *et al.*, 1987). In the crystal structure, there exist  intramolecular  C—H···O, and intermolecular N—H···O and C—H···O hydrogen bonding interactions (Table 1, Fig. 2).

### Experimental

An ethanol solution (50 ml) of 3,4,5-trimethoxybenzohydrazide (0.01 mol) and 4-chlorobenzaldehyde (0.01 mol) was refluxed and stirred for 2 h; the mixture was cooled and the resulting solid product, (I), was collected by filtration. Crystals suitable for single-crystal X-ray diffraction were grown by slow evaporation of a solution in THF.

### Refinement

All H atoms were placed geometrically with C—H= 0.93–0.96 Å and N—H = 0.86 Å, and included in the refinement in riding motion approximation with *U*_iso_(H) = 1.2 or 1.5*U*_eq_ of the carrier atom.

### Crystal data

**Table:**

C_17_H_17_ClN_2_O_4_	*Z* = 2
*M**_r_* = 348.78	*F*_000_ = 364
Triclinic, *P*1	*D*_x_ = 1.418 Mg m^−^^3^
Hall symbol: -P 1	Mo *K*α radiation λ = 0.71073 Å
*a* = 5.119 (2) Å	Cell parameters from 2557 reflections
*b* = 8.210 (4) Å	θ = 2.6–28.3º
*c* = 20.276 (9) Å	µ = 0.26 mm^−^^1^
α = 101.055 (7)º	*T* = 273 (2) K
β = 92.362 (7)º	Block, yellow
γ = 101.459 (7)º	0.12 × 0.10 × 0.06 mm
*V* = 816.9 (7) Å^3^	

### Data collection

**Table:**

Bruker APEX CCD area-detector diffractometer	2860 independent reflections
Radiation source: fine-focus sealed tube	2497 reflections with *I* > 2σ(*I*)
Monochromator: graphite	*R*_int_ = 0.017
*T* = 273(2) K	θ_max_ = 25.0º
φ and ω scans	θ_min_ = 2.1º
Absorption correction: multi-scan(SADABS; Sheldrick, 1996)	*h* = −6→6
*T*_min_ = 0.970, *T*_max_ = 0.985	*k* = −9→9
4307 measured reflections	*l* = −18→24

### Refinement

**Table:**

Refinement on *F*^2^	Hydrogen site location: inferred from neighbouring sites
Least-squares matrix: full	H-atom parameters constrained
*R*[*F*^2^ > 2σ(*F*^2^)] = 0.046	*w* = 1/[σ^2^(*F*_o_^2^) + (0.0602*P*)^2^ + 0.4783*P*] where *P* = (*F*_o_^2^ + 2*F*_c_^2^)/3
*wR*(*F*^2^) = 0.128	(Δ/σ)_max_ < 0.001
*S* = 1.03	Δρ_max_ = 0.59 e Å^−^^3^
2860 reflections	Δρ_min_ = −0.39 e Å^−^^3^
218 parameters	Extinction correction: SHELXL97 (Sheldrick, 2008), Fc^*^=kFc[1+0.001xFc^2^λ^3^/sin(2θ)]^-1/4^
Primary atom site location: structure-invariant direct methods	Extinction coefficient: 0.060 (5)
Secondary atom site location: difference Fourier map	

### Special details

**Table:**

Geometry. All e.s.d.'s (except the e.s.d. in the dihedral angle between two l.s. planes) are estimated using the full covariance matrix. The cell e.s.d.'s are taken into account individually in the estimation of e.s.d.'s in distances, angles and torsion angles; correlations between e.s.d.'s in cell parameters are only used when they are defined by crystal symmetry. An approximate (isotropic) treatment of cell e.s.d.'s is used for estimating e.s.d.'s involving l.s. planes.
Refinement. Refinement of *F*^2^ against ALL reflections. The weighted *R*-factor *wR* and goodness of fit *S* are based on *F*^2^, conventional *R*-factors *R* are based on *F*, with *F* set to zero for negative *F*^2^. The threshold expression of *F*^2^ > σ(*F*^2^) is used only for calculating *R*-factors(gt) *etc*. and is not relevant to the choice of reflections for refinement. *R*-factors based on *F*^2^ are statistically about twice as large as those based on *F*, and *R*- factors based on ALL data will be even larger.

### Fractional atomic coordinates and isotropic or equivalent isotropic displacement parameters (Å^2^)

**Table:**

	*x*	*y*	*z*	*U*_iso_*/*U*_eq_	
Cl1	0.98386 (15)	−0.10593 (8)	0.26691 (3)	0.0637 (2)	
O1	0.5118 (3)	0.4117 (2)	0.66278 (7)	0.0510 (4)	
O2	0.5848 (4)	0.5136 (2)	0.92020 (8)	0.0634 (5)	
O3	0.9795 (4)	0.7795 (3)	0.95374 (9)	0.0883 (7)	
O4	1.3253 (4)	0.8754 (2)	0.86389 (9)	0.0703 (6)	
N1	0.8782 (3)	0.3395 (2)	0.57654 (8)	0.0396 (4)	
N2	0.9449 (3)	0.4260 (2)	0.64227 (8)	0.0393 (4)	
H2	1.1099	0.4630	0.6572	0.047*	
C1	0.8360 (4)	0.5329 (2)	0.75470 (9)	0.0365 (4)	
C2	0.6692 (4)	0.4815 (3)	0.80214 (10)	0.0408 (5)	
H2A	0.5169	0.3961	0.7888	0.049*	
C3	0.7298 (4)	0.5574 (3)	0.86935 (10)	0.0457 (5)	
C4	0.9504 (5)	0.6916 (3)	0.88879 (11)	0.0520 (6)	
C5	1.1177 (4)	0.7419 (3)	0.84062 (11)	0.0480 (5)	
C6	1.0627 (4)	0.6603 (3)	0.77344 (10)	0.0412 (5)	
H6	1.1771	0.6909	0.7414	0.049*	
C7	0.3631 (6)	0.3744 (4)	0.90386 (13)	0.0673 (7)	
H7A	0.2345	0.3994	0.8736	0.101*	
H7B	0.2821	0.3549	0.9443	0.101*	
H7C	0.4225	0.2748	0.8827	0.101*	
C8	1.2015 (8)	0.7925 (6)	0.99320 (16)	0.1086 (14)	
H8A	1.2011	0.6860	1.0061	0.163*	
H8B	1.2073	0.8791	1.0328	0.163*	
H8C	1.3554	0.8220	0.9690	0.163*	
C9	1.5185 (5)	0.9198 (3)	0.81913 (13)	0.0563 (6)	
H9A	1.5826	0.8211	0.7990	0.084*	
H9B	1.6651	1.0047	0.8435	0.084*	
H9C	1.4392	0.9639	0.7845	0.084*	
C10	0.7487 (4)	0.4520 (2)	0.68292 (10)	0.0363 (4)	
C11	1.0749 (4)	0.3316 (3)	0.54076 (10)	0.0421 (5)	
H11	1.2448	0.3915	0.5585	0.050*	
C12	1.0395 (4)	0.2302 (3)	0.47225 (10)	0.0378 (4)	
C13	1.2396 (5)	0.2560 (3)	0.42946 (11)	0.0527 (6)	
H13	1.3898	0.3424	0.4438	0.063*	
C14	1.2205 (5)	0.1556 (3)	0.36576 (11)	0.0542 (6)	
H14	1.3553	0.1749	0.3371	0.065*	
C15	1.0009 (5)	0.0277 (3)	0.34555 (10)	0.0433 (5)	
C16	0.7968 (5)	−0.0006 (3)	0.38638 (12)	0.0540 (6)	
H16	0.6471	−0.0871	0.3717	0.065*	
C17	0.8181 (4)	0.1019 (3)	0.44969 (11)	0.0488 (5)	
H17	0.6803	0.0841	0.4777	0.059*	

### Atomic displacement parameters (Å^2^)

**Table:**

	*U*^11^	*U*^22^	*U*^33^	*U*^12^	*U*^13^	*U*^23^
Cl1	0.0910 (5)	0.0539 (4)	0.0397 (3)	0.0153 (3)	0.0094 (3)	−0.0077 (3)
O1	0.0337 (8)	0.0722 (11)	0.0394 (8)	0.0075 (7)	0.0002 (6)	−0.0034 (7)
O2	0.0650 (11)	0.0788 (12)	0.0361 (9)	−0.0015 (9)	0.0168 (7)	0.0009 (8)
O3	0.0691 (12)	0.1269 (19)	0.0400 (10)	−0.0058 (12)	0.0062 (9)	−0.0264 (11)
O4	0.0600 (11)	0.0767 (12)	0.0477 (10)	−0.0183 (9)	0.0098 (8)	−0.0190 (8)
N1	0.0417 (9)	0.0440 (9)	0.0293 (8)	0.0095 (7)	0.0003 (7)	−0.0018 (7)
N2	0.0339 (8)	0.0504 (10)	0.0288 (8)	0.0084 (7)	−0.0004 (6)	−0.0029 (7)
C1	0.0368 (10)	0.0400 (10)	0.0318 (10)	0.0128 (8)	0.0031 (8)	0.0002 (8)
C2	0.0386 (11)	0.0433 (11)	0.0361 (11)	0.0070 (9)	0.0043 (8)	−0.0009 (9)
C3	0.0458 (12)	0.0555 (13)	0.0341 (11)	0.0120 (10)	0.0102 (9)	0.0027 (9)
C4	0.0493 (12)	0.0641 (15)	0.0329 (11)	0.0084 (11)	0.0046 (9)	−0.0106 (10)
C5	0.0415 (11)	0.0534 (13)	0.0393 (11)	0.0042 (10)	0.0022 (9)	−0.0080 (10)
C6	0.0380 (11)	0.0472 (12)	0.0343 (10)	0.0081 (9)	0.0062 (8)	−0.0015 (9)
C7	0.0720 (17)	0.0708 (17)	0.0523 (15)	−0.0019 (14)	0.0221 (13)	0.0100 (13)
C8	0.092 (2)	0.168 (4)	0.0460 (17)	0.008 (2)	−0.0029 (16)	−0.005 (2)
C9	0.0467 (13)	0.0550 (14)	0.0567 (14)	−0.0008 (10)	0.0047 (11)	−0.0024 (11)
C10	0.0349 (10)	0.0385 (10)	0.0331 (10)	0.0072 (8)	0.0017 (8)	0.0020 (8)
C11	0.0402 (11)	0.0479 (12)	0.0333 (10)	0.0037 (9)	0.0022 (8)	0.0024 (9)
C12	0.0421 (11)	0.0393 (10)	0.0314 (10)	0.0108 (8)	0.0033 (8)	0.0037 (8)
C13	0.0483 (13)	0.0572 (14)	0.0417 (12)	−0.0043 (10)	0.0096 (10)	−0.0020 (10)
C14	0.0599 (14)	0.0586 (14)	0.0400 (12)	0.0062 (11)	0.0178 (10)	0.0033 (10)
C15	0.0584 (13)	0.0389 (11)	0.0329 (10)	0.0161 (9)	0.0037 (9)	0.0019 (8)
C16	0.0534 (13)	0.0481 (13)	0.0493 (13)	−0.0013 (10)	0.0046 (10)	−0.0054 (10)
C17	0.0457 (12)	0.0510 (13)	0.0425 (12)	0.0015 (10)	0.0118 (9)	−0.0013 (10)

### Geometric parameters (Å, °)

**Table:**

Cl1—C15	1.742 (2)	C7—H7A	0.9600
O1—C10	1.223 (2)	C7—H7B	0.9600
O2—C3	1.357 (3)	C7—H7C	0.9600
O2—C7	1.419 (3)	C8—H8A	0.9600
O3—C8	1.337 (4)	C8—H8B	0.9600
O3—C4	1.362 (3)	C8—H8C	0.9600
O4—C5	1.361 (3)	C9—H9A	0.9600
O4—C9	1.411 (3)	C9—H9B	0.9600
N1—C11	1.270 (3)	C9—H9C	0.9600
N1—N2	1.379 (2)	C11—C12	1.460 (3)
N2—C10	1.349 (3)	C11—H11	0.9300
N2—H2	0.8600	C12—C17	1.379 (3)
C1—C2	1.383 (3)	C12—C13	1.380 (3)
C1—C6	1.383 (3)	C13—C14	1.381 (3)
C1—C10	1.489 (3)	C13—H13	0.9300
C2—C3	1.380 (3)	C14—C15	1.365 (3)
C2—H2A	0.9300	C14—H14	0.9300
C3—C4	1.395 (3)	C15—C16	1.371 (3)
C4—C5	1.393 (3)	C16—C17	1.381 (3)
C5—C6	1.388 (3)	C16—H16	0.9300
C6—H6	0.9300	C17—H17	0.9300
			
C3—O2—C7	117.98 (18)	O3—C8—H8C	109.5
C8—O3—C4	120.7 (3)	H8A—C8—H8C	109.5
C5—O4—C9	118.23 (18)	H8B—C8—H8C	109.5
C11—N1—N2	114.90 (17)	O4—C9—H9A	109.5
C10—N2—N1	119.35 (16)	O4—C9—H9B	109.5
C10—N2—H2	120.3	H9A—C9—H9B	109.5
N1—N2—H2	120.3	O4—C9—H9C	109.5
C2—C1—C6	121.06 (18)	H9A—C9—H9C	109.5
C2—C1—C10	116.43 (18)	H9B—C9—H9C	109.5
C6—C1—C10	122.40 (18)	O1—C10—N2	122.65 (18)
C3—C2—C1	119.68 (19)	O1—C10—C1	121.16 (17)
C3—C2—H2A	120.2	N2—C10—C1	116.20 (17)
C1—C2—H2A	120.2	N1—C11—C12	121.29 (19)
O2—C3—C2	124.6 (2)	N1—C11—H11	119.4
O2—C3—C4	115.37 (19)	C12—C11—H11	119.4
C2—C3—C4	120.01 (19)	C17—C12—C13	118.34 (19)
O3—C4—C5	122.0 (2)	C17—C12—C11	122.11 (18)
O3—C4—C3	118.0 (2)	C13—C12—C11	119.44 (19)
C5—C4—C3	119.73 (19)	C12—C13—C14	121.1 (2)
O4—C5—C6	124.2 (2)	C12—C13—H13	119.4
O4—C5—C4	115.72 (19)	C14—C13—H13	119.4
C6—C5—C4	120.0 (2)	C15—C14—C13	119.0 (2)
C1—C6—C5	119.33 (19)	C15—C14—H14	120.5
C1—C6—H6	120.3	C13—C14—H14	120.5
C5—C6—H6	120.3	C14—C15—C16	121.5 (2)
O2—C7—H7A	109.5	C14—C15—Cl1	119.15 (17)
O2—C7—H7B	109.5	C16—C15—Cl1	119.30 (17)
H7A—C7—H7B	109.5	C15—C16—C17	118.7 (2)
O2—C7—H7C	109.5	C15—C16—H16	120.7
H7A—C7—H7C	109.5	C17—C16—H16	120.7
H7B—C7—H7C	109.5	C12—C17—C16	121.3 (2)
O3—C8—H8A	109.5	C12—C17—H17	119.3
O3—C8—H8B	109.5	C16—C17—H17	119.3
H8A—C8—H8B	109.5		
			
C11—N1—N2—C10	175.22 (19)	O4—C5—C6—C1	−175.8 (2)
C6—C1—C2—C3	−0.3 (3)	C4—C5—C6—C1	2.2 (3)
C10—C1—C2—C3	−176.58 (19)	N1—N2—C10—O1	−5.0 (3)
C7—O2—C3—C2	3.4 (4)	N1—N2—C10—C1	174.86 (16)
C7—O2—C3—C4	−178.0 (2)	C2—C1—C10—O1	33.9 (3)
C1—C2—C3—O2	−178.0 (2)	C6—C1—C10—O1	−142.3 (2)
C1—C2—C3—C4	3.4 (3)	C2—C1—C10—N2	−145.93 (19)
C8—O3—C4—C5	−63.6 (4)	C6—C1—C10—N2	37.9 (3)
C8—O3—C4—C3	122.7 (3)	N2—N1—C11—C12	173.85 (18)
O2—C3—C4—O3	−8.6 (3)	N1—C11—C12—C17	−19.4 (3)
C2—C3—C4—O3	170.1 (2)	N1—C11—C12—C13	164.4 (2)
O2—C3—C4—C5	177.6 (2)	C17—C12—C13—C14	−0.4 (4)
C2—C3—C4—C5	−3.7 (4)	C11—C12—C13—C14	176.0 (2)
C9—O4—C5—C6	−9.0 (4)	C12—C13—C14—C15	−0.8 (4)
C9—O4—C5—C4	172.9 (2)	C13—C14—C15—C16	1.5 (4)
O3—C4—C5—O4	5.4 (4)	C13—C14—C15—Cl1	−176.79 (19)
C3—C4—C5—O4	179.0 (2)	C14—C15—C16—C17	−1.0 (4)
O3—C4—C5—C6	−172.7 (2)	Cl1—C15—C16—C17	177.36 (19)
C3—C4—C5—C6	0.9 (4)	C13—C12—C17—C16	1.0 (3)
C2—C1—C6—C5	−2.5 (3)	C11—C12—C17—C16	−175.3 (2)
C10—C1—C6—C5	173.5 (2)	C15—C16—C17—C12	−0.3 (4)

### Hydrogen-bond geometry (Å, °)

**Table:**

*D*—H···*A*	*D*—H	H···*A*	*D*···*A*	*D*—H···*A*
N2—H2···O1^i^	0.86	2.18	2.943 (3)	147
C8—H8C···O4	0.96	2.26	2.896 (4)	123
C11—H11···O1^i^	0.93	2.43	3.145 (3)	134
C16—H16···O1^ii^	0.93	2.57	3.368 (3)	144

Symmetry codes: (i) *x*+1, *y*, *z*; (ii) −*x*+1, −*y*, −*z*+1.
